# TBL2 Is a Novel PERK-Binding Protein that Modulates Stress-Signaling and Cell Survival during Endoplasmic Reticulum Stress

**DOI:** 10.1371/journal.pone.0112761

**Published:** 2014-11-13

**Authors:** Yoshinori Tsukumo, Satomi Tsukahara, Aki Furuno, Shun-ichiro Iemura, Toru Natsume, Akihiro Tomida

**Affiliations:** 1 Cancer Chemotherapy Center, Japanese Foundation for Cancer Research, Koto-ku, Tokyo, Japan; 2 Biomedicinal Information Research Center, National Institute of Advanced Industrial Science and Technology, Koto-ku, Tokyo, Japan; University of Hong Kong, Hong Kong

## Abstract

Under ER stress, PKR-like ER-resident kinase (PERK) phosphorylates translation initiation factor eIF2α, resulting in repression of global protein synthesis and concomitant upregulation of the translation of specific mRNAs such as activating transcription factor 4 (ATF4). This PERK function is important for cell survival under ER stress and poor nutrient conditions. However, mechanisms of the PERK signaling pathway are not thoroughly understood. Here we identify transducin (beta)-like 2 (TBL2) as a novel PERK-binding protein. We found that TBL2 is an ER-localized type-I transmembrane protein and preferentially binds to the phosphorylated form of PERK, but not another eIF2α kinase GCN2 or ER-resident kinase IRE1, under ER stress. Immunoprecipitation analysis using various deletion mutants revealed that TBL2 interacts with PERK via the N-terminus proximal region and also associates with eIF2α via the WD40 domain. In addition, TBL2 knockdown can lead to impaired ATF4 induction under ER stress or poor nutrient conditions such as glucose and oxygen deprivation. Consistently, TBL2 knockdown rendered cells vulnerable to stresses similarly to PERK knockdown. Thus, TBL2 serves as a potential regulator of the PERK pathway.

## Introduction

The unfolded protein response (UPR) is a survival stress response enabling the cell to cope with the accumulation of unfolded proteins in the endoplasmic reticulum (ER) causing ER stress. Three ER-membrane sensor proteins, PERK, activating transcription factor 6 (ATF6) and inositol-requiring enzyme 1 (IRE1), play important roles in the UPR signaling [1], [2]. These sensor proteins are activated in response to ER stress and transmit the signals to activate both transcriptional and translational gene expression programs. The UPR occurs under such pathophysiological cell conditions as hypoxia, nutrient starvation and low pH. The UPR activation has also been seen in some human diseases, including diabetes, neurodegenerative disease and cancer, and their progressions [1]–[3].

PERK has been known to induce a response that represses general mRNA translation and promotes translation of a subset of mRNAs [4], [5]. During the UPR, PERK is activated by oligomerization and autophosphorylation, and the activated PERK subsequently phosphorylates alpha subunit of eukaryotic initiation factor 2 (eIF2α) at Ser-51, resulting in reducing global translation [4], [5]. Under conditions where eIF2α is phosphorylated, most mRNA translation is suppressed while translation of a particular subset of mRNAs as represented by activating transcription factor 4 (ATF4) is elevated [6]. ATF4 expression is increased in response to ER stress and a variety of tumor microenvironmental stresses including low glucose, hypoxia, amino acid depletion [7]. In tumors, ATF4 expression is detected in hypoxic- and nutrient-deprived regions where it plays an important role in maintaining metabolic homeostasis and promoting cancer cell survival by transcriptionally regulating amino acid uptake and biosynthesis, autophagy, redox balance and angiogenesis [7]. Thus, the PERK-eIF2α-ATF4 axis is well characterized. However, the existence of the additional effectors of the PERK pathway has not been fully addressed. Here we show transducin (beta)-like 2 (TBL2) as a novel PERK-binding protein.

TBL2 is a ubiquitously expressed protein with a predicted transmembrane region, WD40 repeats, and a coiled coil domain [8]. TBL2 has been associated with some disorders like Williams-Beuren syndrome (WBS), in which the *TBL2* gene is typically deleted. Patients with WBS suffer a developmental disorder caused by deletion of 26–28 genes at chromosome 7q11.23 [8], [9]. They exhibit several common features, including cardiovascular abnormality, hypercalcemia, characteristic facial appearance, mental retardation [8]–[10]. Although the cardiovascular abnormality in WBS has been explained by the loss of an elastin (ELN) allele, the phenotypic consequences of losing other alleles, including the *TBL2* gene, are much less clear. The Tbl2 knockout mouse exhibited increased mean body weight, length, and change in bone metabolism [11]. These observations in knockout mice, however, are not necessarily consistent with phenotype in WBS patients. In addition to genetic loss of TBL2, SNP in the human *TBL2* gene has been reported to associate with increased blood triglycerides, a lipidemia marker, although the effect of SNP on TBL2 function is unknown [12], [13]. Thus, TBL2 dysregulation could be involved in several disease phenotypes; however, the cellular and molecular functions of TBL2 remain to be elucidated.

Using mass spectrometry, we identified TBL2 as a novel PERK-interacting protein. Our experiments revealed that TBL2 is a type I ER transmembrane protein and preferentially associates with phospho-PERK. Importantly, TBL2 was involved in induction of ATF4 expression under stress conditions such as glucose/oxygen deprivation and the cell growth. Thus, our results indicate that TBL2 is a new player on the PERK signaling pathway.

## Materials and Methods

### Chemicals and antibodies

2-Deoxyglucose (Sigma, St Louis, MO), histidinol (Sigma) and DTT (Nacalai Tesque, Kyoto, Japan) was dissolved in distilled, sterilized water. Tunicamycin (Nacalai Tesque) and thapsigargin (Wako Pure Chemical Industries, Osaka, Japan) were dissolved in dimethyl sulfoxide. Hydrogen peroxide was purchased from WAKO. These compounds were added to culture medium, with the solvent being less than 0.5% of the medium’s volume. The following commercially available antibodies were used: rabbit anti-TBL2 and anti-ATF4 (ProteinTech, Chicago, IL), anti-PERK, anti-eIF2 alpha (abcam, Cambridge, MA), anti-phospho-PERK (BioLegend, San Diego, CA), anti-phospho-eIF2 alpha (Ser51), anti-calnexin (Cell Signaling Technology, Danvers, MA), anti-KDEL for GRP78 (StressGen, Victoria, BC, Canada) anti-FLAG M2 (Sigma), and HRP- or FITC-conjugated anti-V5 (Invitrogen), HRP-conjugated anti-rabbit or mouse IgG (GE Healthcare Bio-Sciences Corp, Piscataway, NJ).

### Cell lines and treatment

We used following cell lines: Human fibrosarcoma HT1080 cells [14], human renal cell carcinoma 786-O cells [14], human embryonic kidney 293T cells [15] and 293 cells (CRL-1573). HT1080 and 786-O cells were maintained in RPMI 1640 medium and 293 and 293T cells in Dulbecco’s modified Eagle medium (DMEM) supplemented with 10% heat-inactivated fetal bovine serum and 100 µg/mL of kanamycin. All cells were cultured at 37°C in a humidified atmosphere containing 5% CO_2_. Glucose-free RPMI 1640 medium was obtained from Invitrogen (Carlsbad, CA) and supplemented with 10% heat-inactivated fetal bovine serum for experimental use. To create hypoxic conditions, cells were placed in a plastic box with Anaero Pack Kenki for Cell (Mitsubishi Gas Chemical, Tokyo, Japan).

### Plasmids

pFLAG-PERK plasmids were constructed by inserting full-length PERK into the p3XFLAG-CMV-14 or pFLAG-cmv 5c vector (Sigma) at the KpnI site. pFLAG-PERK-DN plasmid was constructed by inserting PERK lacking kinase domain into pFLAG-cmv 5c vector (Sigma) at the KpnI site. pFLAG-TBL2 WT or each mutant and pFLAG-IRE-1 were constructed by ligating each cDNA amplified by RT-PCR at the HindIII/NotI site into the pFlag-CMV-5c vector (Sigma). V5-tagged TBL2 was constructed by ligating cDNA amplified by RT-PCR into pcDNA3.1 TOPO V5/His (Invitrogen). pShooter pCMV/Myc/ER/GFP (Invitrogen), which produces GFP with the N-terminal ER signal peptide and the C-terminal ER retention signal sequence, was used as an ER marker. Transient transfections were performed using Lipofectamine 2000 (Invitrogen) or lipofectamine RNAi MAX (Invitrogen), according to the manufacturer’s protocol.

### siRNAs

Stealth siRNAs against TBL2 (#1: HSS146815, #2: HSS146816) or PERK (#1: HSS114059, #2: HSS114060) were purchased from Invitrogen. For transient transfection of siRNA, cells were seeded at a density of 4×10^5^/well in a type I, collagen-coated, 6-well plate and were cultured overnight. The cells were transfected for 6 h with siRNA (10 nM), using the Lipofectamine RNAi MAX reagent according to the manufacturer’s protocol. Then, the cells were reseeded from 1 well to 3 wells. Two days after transfection, the cells were used for experimentation. Sequences are TBL2 #1∶5′- UCU UCU UGU AUU CCA CAU CUG UGU C −3′, 5′- GAC ACA GAU GUG GAA UAC AAG AAG A-3′; TBL2 #2∶5′- UCA UCU UGA AGA CAC GGA GGG UGU C −3′, 5′- GAC ACC CUC CGU GUC UUC AAG AUG A −3′; PERK #1∶5′- UUU ACU GUG AAG AAA CUC CAC UGC C-3′, 5′- GGC AGU GGA GUU UCU UCA CAG UAA A-3′; PERK #2∶5′- AAU ACC UCU GGU UUG CUA AGG CUG G-3′, 5′- CCA GCC UUA GCA AAC CAG AGG UAU U-3′.

### Mass spectrometry

HEK293T cells were transfected with a pFLAG-PERK plasmid and the cell lysate was immunoprecipitated with a FLAG antibody. PERK-binding proteins were analyzed by direct nano-flow liquid chromatography/electrospray tandem mass spectrometry, as described earlier [16].

### Immunoblot analysis

Immunoblot analysis was performed as described previously [15]. Briefly, cells were lysed in 1×SDS sample buffer, and protein concentrations of the lysates were measured with a BIO-RAD protein assay kit (Bio-Rad, Hercules, CA). Equal amounts of proteins were resolved on a 10% SDS-polyacrylamide gel and transferred by electroblotting to a nitrocellulose membrane. Membranes were probed with antibodies, as indicated, and the specific signals were detected using an enhanced chemiluminescence detection system (GE Healthcare Bio-Sciences Corp., Tokyo, Japan).

### Immunoprecipitation

Immunoprecipitation was performed as described previously [15]. Briefly, cells were washed with ice-cold PBS and lysed in 50 mM Tris-HCl (pH 8.0), 1% Triton X-100, 150 mM NaCl, 1 mM EDTA supplemented with protease inhibitors and a phosphatase inhibitor cocktail (Sigma). The lysates were cleared by centrifugation at 13,000×g for 10 min at 4°C and immunoprecipitated by anti-FLAG- or anti-V5-conjugated beads (Sigma) in lysis buffer. Immunoprecipitates were prepared for immunoblot analysis by washing three times with lysis buffer and eluting by 3× FLAG peptide (Sigma) or boiling in SDS sample buffer.

### Immunofluorescence

Cells on a poly-lysine-coated cover slip were fixed and permeabilized for 10 min in PBS-containing 4% paraformaldehyde and 0.1% Triton X-100. After blocking for 1 h in PBS with 10% BSA, the cells were incubated with primary (mouse anti-FLAG M2, 1∶1000; anti-FLAG M2, 1∶3000, rabbit anti-V5 or rabbit anti-GFP, 1∶1000 (Invitrogen)) and subsequent secondary antibodies (Alexa-fluor 488–conjugated anti-mouse Ig (1∶1000), or Alexa-fluor 568–conjugated anti-rabbit Ig (1∶1000) (Invitrogen)) in PBS with 1.5% BSA for 1 h at room temperature. The cover slips were mounted on microscope slides. Fluorescence images were obtained with an Olympus Fluoview 500 confocal microscope using 488-nm laser excitation for Alexa-488 or 543-nm for Alexa-568.

### Subcellular fractionation

Subcellular fractionation was modified according to a previously reported procedure [17]. 293T cells were harvested, disrupted in buffer A (50 mM Tris-Hcl (pH7.5), 5 mM EDTA, 1 mM DTT) using a Dounce-type homogenizer and then centrifuged at 1000×g for 10 min to obtain the nuclear pellet (N). The resulting supernatant was mixed with the same volume of 880 mM sucrose-containing buffer A and then centrifuged at 100,000×g for an additional 1 h to separate the soluble cytosolic fraction (C) in suspension from mitochondria and microsome fraction (M) in pellet.

### Trypsin digestion assay

Trypsin digestion assay was modified according to a previously reported procedure [17]. 293T cells were transfected with pTBL2 (C-terminal V5-tag) and then the cells were disrupted in a buffer (50 mM Tris-Hcl (pH7.5), 5 mM EDTA, 1 mM DTT) using a Dounce-type homogenizer. The nucleus was removed by centrifugation, and then the remaining crude cell extract, which contained microsome [M] and cytosol [C] fractions, was digested with 0.25% trypsin solution for 5 min at room temperature. The reaction was terminated by adding equal volume of 4% SDS buffer, and each sample was subjected to immunoblot analysis.

### [^35^S]Methionine incorporation assay

293 cells were transfected transiently with stealth siRNA (4×10^5^/well in a 6-well plate). After 48 h, cells were incubated for 5 min in Met-Cys-free DMEM supplemented with 2 mM glutamine, 10% dialyzed FBS. After pretreatment with thapsigargin for 20 min, the cells were labeled for 20 min using the 100 µCi/ml easy tag EXPRES ^35^S protein labeling mix (PerkinElmer, Waltham, MA) in the presence of thapsigargin. Then, each sample was boiled for 5 min in SDS buffer and subjected to SDS-PAGE. After gel drying, the incorporated [^35^S]Met/Cys was visualized with the Typhoon9410 (GE Healthcare).

### Establishment of shRNA-expressing cells

Lentivirus particles encoding individual shRNA (SHC002 shRNA Control, TRCN0000323118, 0000323032, 0000323108, 0000323235 against TBL2, TRCN0000262374 against PERK) were purchased from Sigma-Aldrich. Stable shRNA-expressing 786-O cells were established according to the manufacturer’s protocol and were selected using puromycin.

### Measurement of cell growth and viability

Control-, TBL2- and PERK-shRNA-expressing 786-O cells were incubated for 12 h under glucose- or O_2_-deprived conditions or under both. Immediately thereafter, cells were reseeded onto 12-well plates and incubated in normal culture medium for 3 days. Relative cell numbers after 3 days were measured using an MTT assay. To analyze viability of cells treated with thapsigargin, intracellular ATP level was measured by using a commercial kit (promega: Cell Titer-Glo Luminescent Cell Viability Assay). For cell proliferation assay, cells were reseeded to 6-well plate after incubation for 12 h under glucose- and/or O_2_-deprived conditions. The cell numbers at each time point (1–4 days) were measured automatically using a Beckman Coulter Counter (Brea, CA).

### Statistical analyses

Statistical analysis was performed using student's t-test. We considered a P-value of <0.05 statistically significant.

## Results

### TBL2 is an ER-localized type-I transmembrane protein

**Figure 1:**
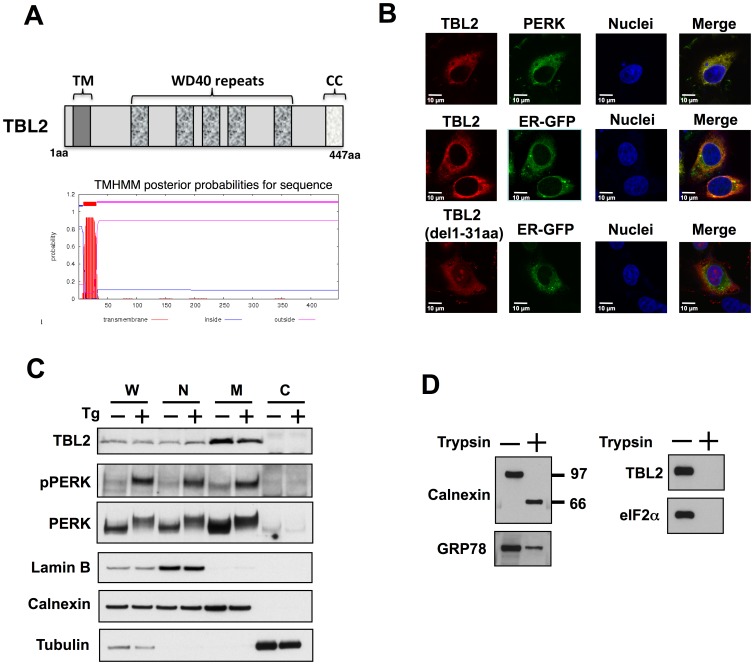
TBL2 is an ER-localized type-I transmembrane protein. (A) The TBL2 domain structure was depicted by the SMART protein domain prediction program. TM: transmembrane region, WD40 domain, CC: coiled coil domain. bottom, topology of the TBL2 protein. Transmembrane domain prediction was done by using the TMHMM algorithm. (B) HT1080 cells were transiently transfected with each plasmid and then were fixed and analyzed by immunofluorescence using a confocal microscope. (C) Distribution of TBL2 in fractions of 293T cells. The cells were disrupted using a Dounce-type homogenizer and then each fraction (whole lysate [W], nuclei [N], mitochondria and ER [M], cytoplasm [C]) was separated by centrifugation and subjected to immunoblot analysis. (D) 293T cells were transfected with pTBL2 (V5-tag) and then the cells were disrupted using a Dounce-type homogenizer. The nucleus was removed by centrifugation and then the remaining crude cell extract, which contains microsome [M] and cytosol [C] fractions, was digested with trypsin for 5 min and subjected to immunoblot analysis.

To address the molecular mechanisms of the PERK signaling pathway, we screened novel PERK-binding partners by analyzing PERK-coprecipitated proteins in transiently PERK-overexpressed 293T cells using direct nano-flow liquid chromatography/tandem mass spectrometry [16]. As a result, we identified TBL2 (also termed WS-beta-TRP, WBSCR13), the function of which is unknown [8]. The SMART protein domain prediction program (http://smart.embl.de/) suggested that TBL2 contained the N-terminal proximal transmembrane region (TM: 9-31aa), the WD40, and the C-terminal coiled coil domains (Figure 1A). In general, the WD40 and the coiled coil domains are known to engage in protein-protein interaction. In addition, hydropathy analysis of TBL2 with the TMHMM algorithm (http://www.cbs.dtu.dk/services/TMHMM/) predicted one transmembrane domain corresponding to 9-31aa (Figure 1A, bottom). We examined the subcellular localization of TBL2 and found it in the ER, as shown by co-localization with PERK and ER-GFP (GFP with an ER localization signal, see Materials and Methods). In contrast, the del 1-31aa TBL2 mutant that lacked the putative TM region exhibited a broadly diffused staining pattern in the cell, suggesting that the TM region functions as ER-anchor region (Figure 1B). We further analyzed the intracellular distribution pattern of TBL2 in a cell fractionation experiment. Consistent with type I ER transmembrane proteins PERK and calnexin, TBL2 was enriched in the M fraction, which contains the ER and mitochondria, under both thapsigargin-treated or non-treated conditions (Figure 1C). The TBL2 protein, as well as PERK and calnexin, was also recovered in the N fraction probably because nuclear outer membrane is contiguous with ER membrane (Figure 1C).

To determine the membrane orientation of TBL2, we examined its sensitivity to trypsin digestion using crude cell extracts that contained microsome and cytoplasm (Figure 1D). TBL2, as well as cytoplasmic protein eIF2α, disappeared completely after trypsin treatment, while the ER lumenal segment of calnexin or the ER lumenal protein GRP78 was protected from trypsin digestion (Figure 1D). These results strongly suggest that TBL2 localized in the ER via the TM region and that the C-terminal segment (32-447aa) of TBL2 faces the cytoplasm, that is, TBL2 is a type I ER transmembrane protein.

### TBL2 interacts with PERK in response to ER stress

**Figure 2:**
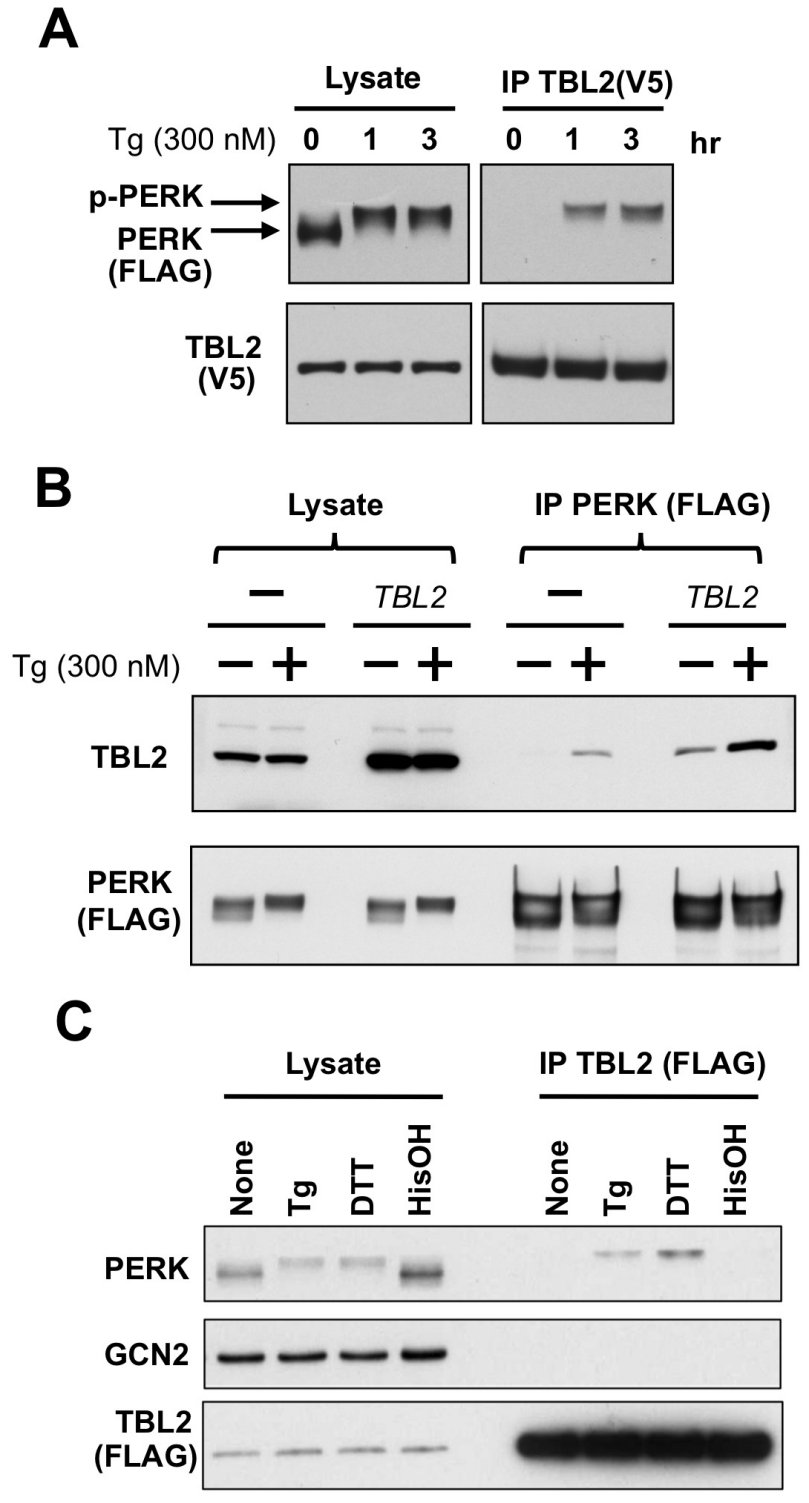
TBL2 interacts with PERK in response to ER stress. (A) 293T cells were transiently co-transfected with pTBL2 (V5-tag) and pFLAG-PERK, and then were treated with 300 nM thapsigargin for 1, or 3 h. The cell lysates were immunoprecipitated with anti-V5 antibody and immunoblotted with anti-FLAG or anti-V5 antibody. (B) 293T cells were transiently transfected with pFLAG-PERK or together with pTBL2 (non-tag). After immunoprecipitation with anti-FLAG conjugated beads, PERK-bound TBL2 protein was detected with anti-TBL2 antibody. (C) 293T cells were transiently transfected with pFLAG-TBL2 and then were treated with 300 nM thapsigargin for 1 hour, 1 mM DTT for 30 min or with 5 mM histidinol (HisOH) for 4 hour. After immunoprecipitation with anti-FLAG antibody-conjugated beads, each protein was immunoblotted with the indicated antibody.

To confirm binding of TBL2 to PERK, we performed immunoprecipitation and immunoblotting after cotransfection of PERK and TBL2 plasmids into 293T cells. As shown in Figure 2A, PERK coprecipitated with TBL2 when cells were treated with thapsigargin, a representative ER stress-inducing agent. Similarly, immunoprecipitation of PERK protein also showed thapsigargin-stimulated binding of endogenous or exogenous TBL2 protein (Figure 2B). The PERK-TBL2 interaction was also stimulated by treatment with other ER stress inducer DTT, but not amino acid starvation-mimicking agent histidinol [18] (Figure 2C). In contrast to PERK, TBL2 did not interact with GCN2, another eIF2α kinase (Figure 2C).

### Preferential binding of TBL2 to phospho-PERK

**Figure 3:**
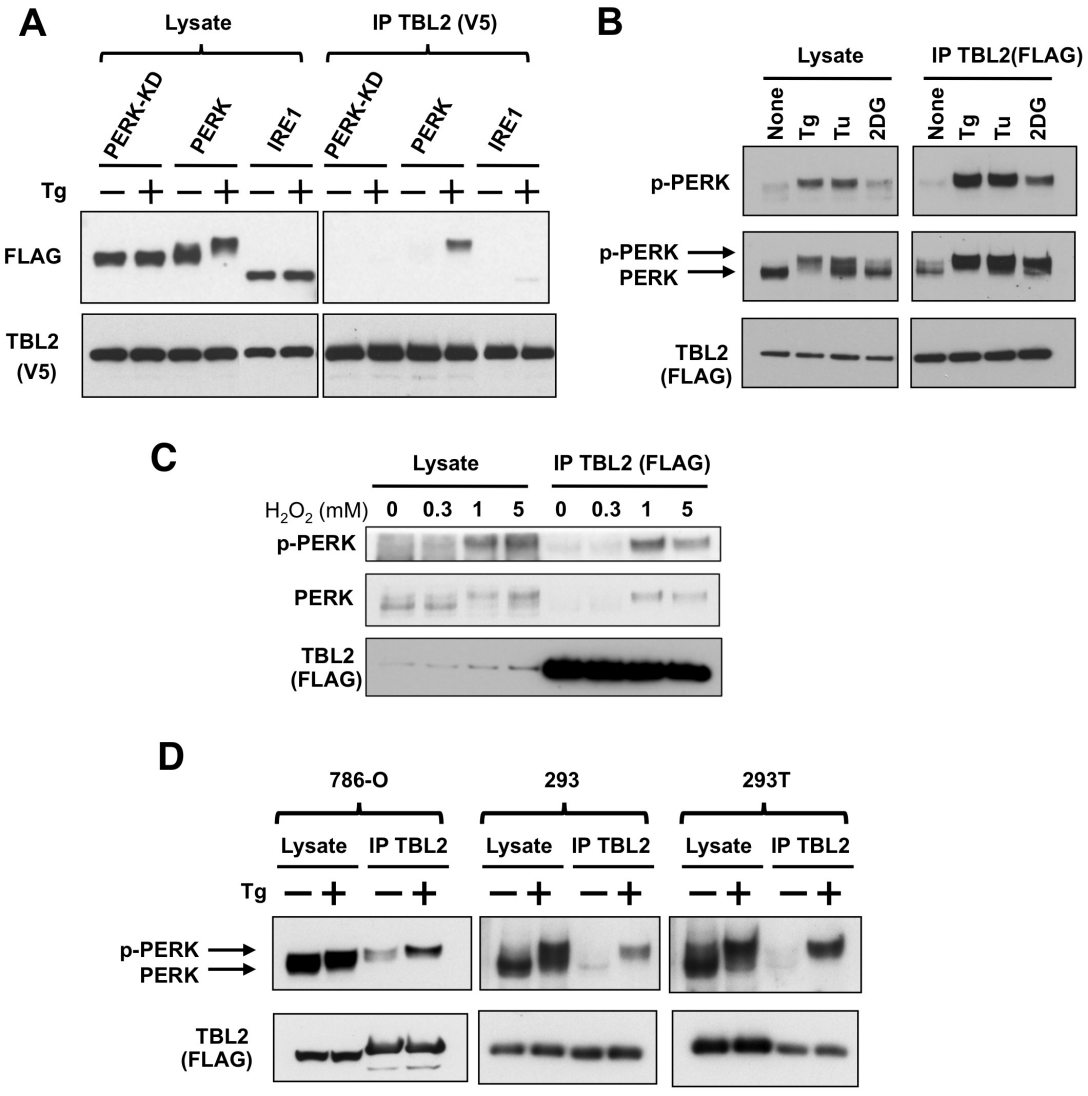
Preferential binding of TBL2 to phospho-PERK. (A) 293T cells were transiently co-transfected with pTBL2 (V5-tag) and either pFLAG-PERK, pFLAG-PERK(K621A) or pFLAG-IRE1 and then were treated with 300 nM thapsigargin (Tg) for 2 h. The cell lysates were immunoprecipitated with anti-V5 antibody and immunoblotted with anti-FLAG or anti-V5 antibody. (B) 293T cells were transiently transfected with pFLAG-TBL2 and then were treated with 300 nM thapsigargin (Tg), 4 µg/ml tunicamycin (Tu) or 10 mM 2-deoxyglucose (2DG) for 2 h. Endogenous PERK protein was detected with anti-PERK or anti–phospho-PERK antibody. (C) 293T cells were transiently transfected with pFLAG-TBL2 and then were treated with the indicated doses of hydrogen peroxide (H_2_O_2_) for 4 hour. After immunoprecipitation with anti-FLAG antibody-conjugated beads, each protein was immunoblotted with the indicated antibody. (D) 786-O, 293 and 293T cells were transiently transfected with pFLAG-TBL2 and then were treated with 300 nM thapsigargin (Tg) for 1 hour. After immunoprecipitation with anti-FLAG antibody-conjugated beads, each protein was immunoblotted with the indicated antibody.

As seen in Figure 2, the electrophoretic mobility of the coprecipitated PERK protein corresponded with its autophosphorylated form. To verify whether TBL2 preferentially interacted with phospho-PERK, we investigated the interaction with the PERK kinase-dead form K621A (PERK-KD) or another type-I ER transmembrane kinase, IRE1, which is another important sensor of the UPR [1], [2]. As shown in Figure 3A (right panel), immunoprecipitation showed that PERK was dominantly detected in thapsigargin-dependent manner while PERK-KD or IRE1 was not or only faintly detected. Moreover, the interaction with phospho-PERK was confirmed using phospho-specific antibody (Figure 3B). The interaction was also induced by several kinds of ER stress-inducing agents including thapsigargin, tunicamycin, 2-deoxy-glucose, hydrogen peroxide and observed in several cell lines (Figure 3B–D). Thus, TBL2 specifically interacted with phospho-PERK in response to ER stress.

### Identification of PERK- and eIF2α-binding region

**Figure 4:**
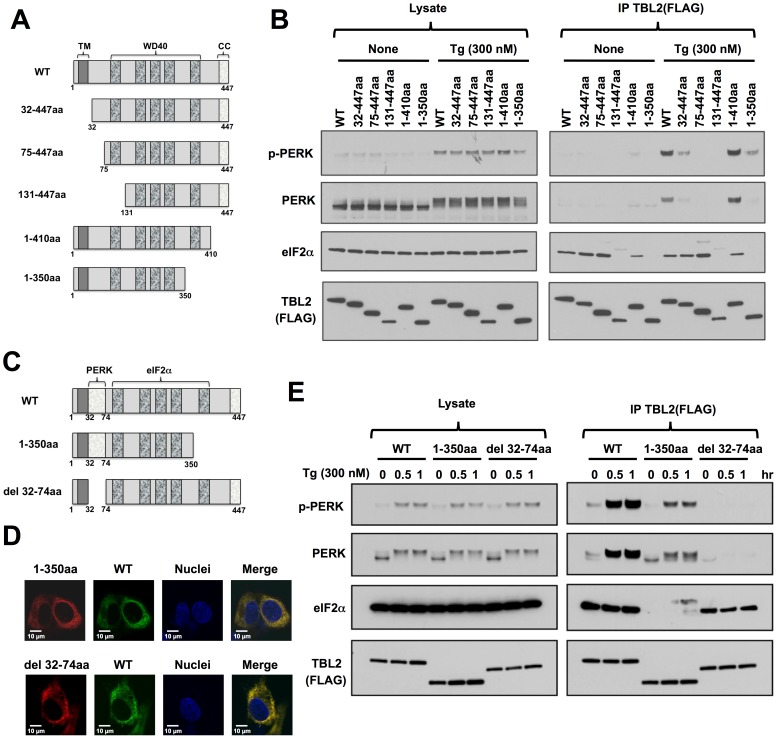
Identification of PERK- and eIF2α-binding region. (A) Schematic representations of each TBL2 mutant. (B) 293T cells were transiently transfected with each TBL2 mutant plasmid and then treated with 300 nM thapsigargin for 1 h. After immunoprecipitation, each sample was subjected to immunoblot analysis. (C) Schematic representations of each TBL2 mutant. (D) HT1080 cells were transiently co-transfected with pTBL2 mutant (FLAG, red) and pTBL2 WT (V5-tag, green) plasmids. After 24 hours, cells were fixed and then analyzed by immunofluorescence using confocal microscope. (E) 293T cells were transiently transfected with each TBL2 mutant plasmid and then treated with 300 nM thapsigargin for 0.5 or 1 h. After immunoprecipitation, each sample was subjected to immunoblot analysis.

We constructed a number of TBL2 deletion mutants to determine which regions would be required for interaction with PERK (Figure 4A). In this analysis, we also examined whether TBL2 interacts with eIF2α because it is a well-characterized PERK substrate [4], [5]. Interestingly, TBL2 also associated with eIF2α under both normal and thapsigargin-treated conditions (Figure 4B right panel, lanes with “WT”). The mutants that lacked part of the WD40 domain, 131-447aa and 1-350aa, completely lost the ability to associate with eIF2α (Figure 4B), suggesting that a large region of the WD40 domain is required for interaction with eIF2α. Similar requirement of large region of WD40 domain for proper activity has been shown in previous reports on WD40 proteins, UAF1 and COP1 [19], [20]. Given that the WD40 domain forms a circularized, propeller structure consisting of each blade of WD40 repeats [21], all of the WD40 repeats may be required for correct folding of TBL2. Next, we found that the 32-447aa TBL2 mutant lacking the N-terminal TM region exhibited impaired interaction with phospho-PERK, probably due to its inability to be retained in the ER membrane (Figure 4B and 1B). The 75-447aa mutant completely lost the PERK interaction ability, suggesting that 32-74aa was crucial for phospho-PERK binding (Figure 4B). The C-terminal deletion of TBL2 (the 1-350aa mutant) also weakened the interaction with PERK but the mutant was still able to bind to PERK (Figure 4B). To determine which region binds to phospho-PERK, we constructed the del32-74aa mutant, which lacked the 32-74aa region only, and compared it with the 1-350aa (Figure 4C). Both mutants localized in the ER (Figure 4D). The del32-74aa mutant kept the association with eIF2α but completely lost the ability to interact with phospho-PERK (Figure 4E). In contrast, while the 1-350aa mutant could not associate with eIF2α (Figure 4E), it still had phospho-PERK binding ability (Figure 4E). Therefore, we concluded that TBL2 interacts with phospho-PERK via the 32-74aa region and also associates with eIF2α via the WD40 domain. Thus, TBL2 forms the complex via its distinct regions. In addition, a PERK mutant lacking its cytoplasmic region (PERK-DN) barely bound to TBL2 despite a greater expression levels than those of PERK-WT (Figure S1), suggesting that TBL2 interacts likely with the cytoplasmic region of PERK.

### TBL2 knockdown impairs ATF4 induction under stress conditions

**Figure 5:**
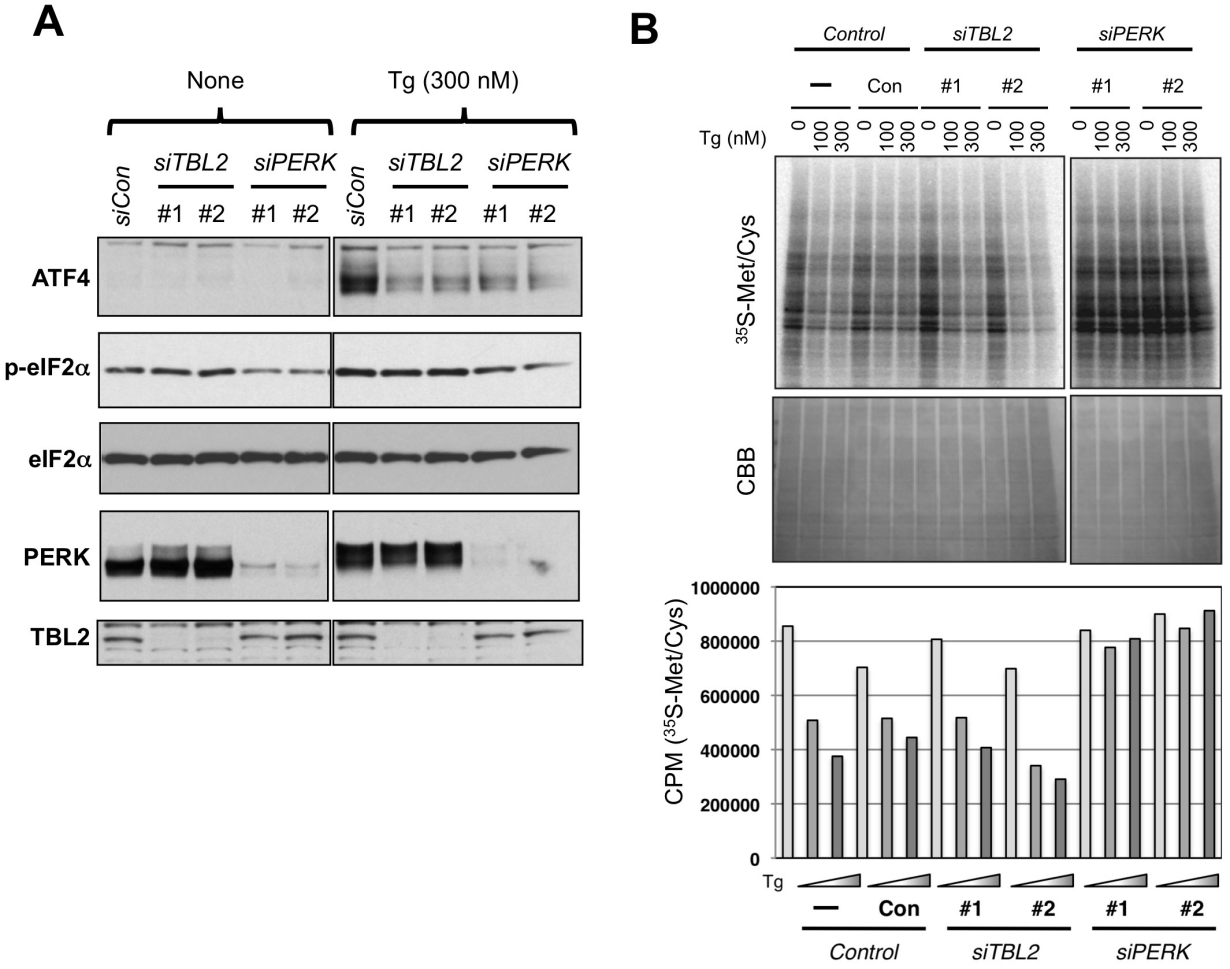
Effects of TBL2 knockdown on the PERK pathway. (A) 293 cells were transiently transfected with non-silencing siRNA, two TBL2 siRNAs (#1, #2) or PERK siRNAs (#1, #2). After 48 h, the cells were treated with 300 nM thapsigargin for 90 min and analyzed by immunoblot anaysis. (B) 293 cells were transiently transfected with non-silencing siRNA, two TBL2 siRNAs (#1, #2) or PERK siRNAs (#1, #2). The protein synthesis rate was measured by incorporating [^35^S]methionine/cysteine. The pulse labeling was carried out during the last 20 min of the 40-min thapsigargin (Tg) treatment (100 or 300 nM). Upper: autoradiography image of SDS-PAGE. Lower panel: TCA precipitation sample was measured using a scintillation counter.

Next, we examined whether TBL2 plays a role in the PERK signaling pathway. For this purpose, we conducted knockdown analysis using siRNA against TBL2. In control siRNA transfected cells, eIF2α phosphorylation and ATF4 expression were induced in a thapsigargin-dependent manner while PERK knockdown reduced the eIF2α phosphorylation and the following ATF4 protein induction (Figure 5A). TBL2 knockdown also impaired ATF4 induction at the similar level to PERK knockdown; however, it did not affect the stress-induced eIF2α phosphorylation (Figure 5A). Next, we examined whether TBL2 knockdown had an effect on global protein synthesis since PERK-mediated eIF2α phosphorylatioin leads to translational repression [4], [5]. As assessed by [^35^S]Met/Cys radiolabeling, thapsigargin treatment clearly reduced protein synthesis in control or TBL2 knockdown cells but not in PERK knockdown cells (Figure 5B top and bottom). Thus, TBL2 was unlikely involved in either eIF2α phosphorylation or general translational repression upon ER stress.

We also investigated the effects of TBL2 knockdown on XBP1 splicing and GRP78 induction, which are representative downstream indicators of activation of IRE1 and ATF6 pathways, respectively (Figure S2A and S2B) [22], [23]. Both XBP1 splicing and GRP78 induction occurred in TBL2 knockdown cells at a similar level to control cells. Thus, TBL2 appears to be a selective regulator of the PERK pathway.

### TBL2 plays an important role in cell growth after exposure to glucose and oxygen deprivation

**Figure 6:**
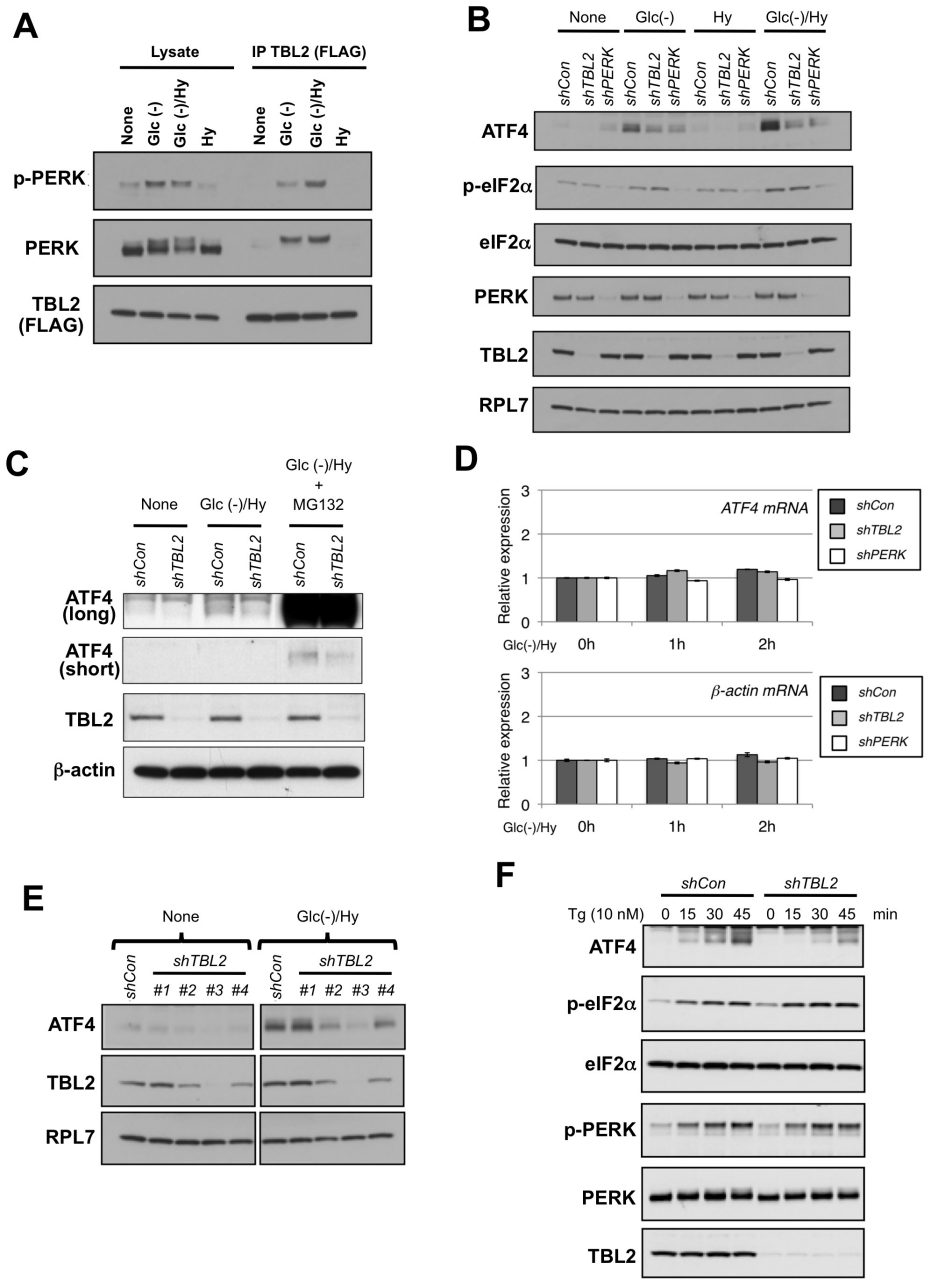
TBL2 knockdown impairs ATF4 induction under glucose- and oxygen-deprived conditions. (A) 293T cells were transiently transfected with pFLAG-TBL2. Then, the cells were incubated for 4 h under glc(−) and/or hypoxic conditions (Hy). After immunoprecipitation with anti-FLAG-conjugated beads, each sample was subjected to immunoblot analysis. (B) Control-, TBL2- or PERK-shRNA-expressing 786-O cells were incubated for 2 h under glc(−) and/or hypoxic conditions (Hy). Each sample was subjected to immunoblot analysis. (C) Control or TBL2 shRNA-expressing cells were exposed to glc(−) and/or hypoxia (Hy) for 2 h in the presence or absence of 10 µM MG132. (D) TBL2- or PERK-shRNA-expressing cells were incubated for the indicated times under glc(−) and hypoxic conditions (Hy). *ATF4* mRNA induction under glc(−)/hypoxic conditions was measured by qRT-PCR. *β-actin* mRNA levels were used for normalization.

We also examined the role of TBL2 under low glucose and hypoxic conditions, which arephysiological cell conditions observed in the tumor microenvironment or during ischemia and that cause the UPR [2], [3], [24], [25]. As expected, glucose withdrawal (glc(−)) induced a PERK-TBL2 interaction (Figure 6A). Although hypoxia alone did not trigger the interaction at this time point (4 h), hypoxia combined with glc(−) enhanced PERK-TBL2 interaction compared with glc(−) alone (Figure 6A). We examined the response to glc(−) and hypoxia using stably TBL2-shRNA-expressing cells. In control shRNA-expressing cells, the glc(−)/hypoxia combination stimulated a ATF4 protein expression more strongly than each stressor alone (Figure 6B). By contrast, but similarly to the results of siRNA experiments (Figure 5), ATF4 induction in TBL2-shRNA-expressing cells was impaired and the eIF2α phosphorylation was similar level to that in control cells (Figure 6B). In addition, impaired ATF4 induction in TBL2 knockdown cells was observed even in the presence of a proteasome inhibitor MG132 (Figure 6C), suggesting that TBL2 is unlikely involved in protein degradation of ATF4. In contrast to decrease in the expression at the protein level, ATF4 mRNA expression was largely unchanged in TBL2-shRNA-expressing cells compared to control cells (Figure 6D). Thus, TBL2 appears to mediate the post-transcriptional process of ATF4 expression under glc(−)/hypoxia.

**Figure 7:**
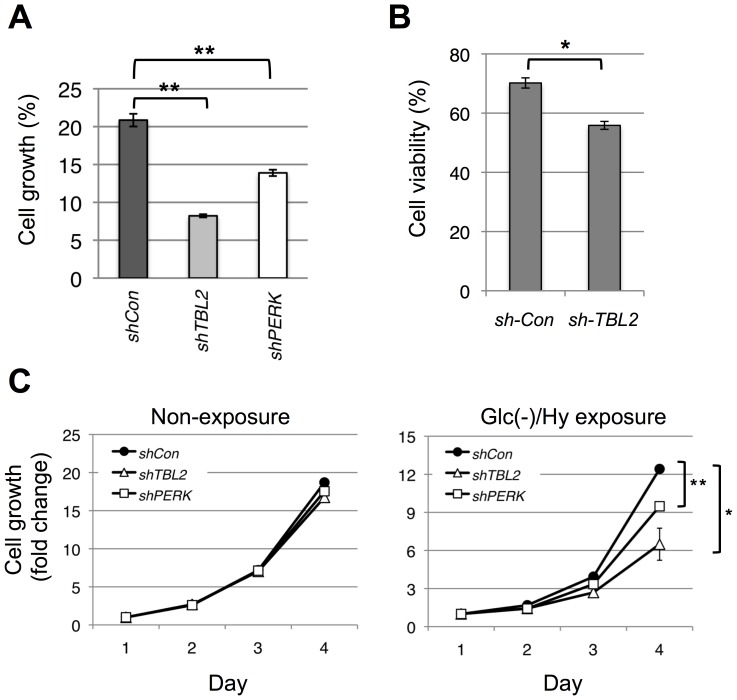
TBL2 plays an important role in cell growth after exposure to glocose and oxygen deprivation. (A) TBL2 or PERK shRNA-expressing cells were exposed to glc(−) and/or hypoxia for 12 h and then the cells were reseeded. Relative cell numbers after 3 days were measured by MTT assay. Data is representative of three independent experiments (n = 3) *P<0.05; **P<0.01. (B) Control or TBL2 shRNA-expressing cells were treated with 2 µM thapsigargin for 24 h. Cell viability was calculated by measuring intracellular ATP level. Data is representative of three independent experiments (n = 3) *P<0.05; **P<0.01. (C) The shRNA-expressing cells were exposed to glc(−) and hypoxia for 12 h and were then immediately reseeded. Then the cells were incubated in normal medium for the indicated time (days). Cell numbers at the each time point were counted automatically using a Beckman Coulter Counter. Data is representative of three independent experiments (n = 3) *P<0.05; **P<0.01.

We further investigated growth or viability of TBL2-shRNA-expressing cells exposed to stress using several assays, including MTT assay (Figure 7A), ATP-based cell viability assay (Figure 7B) and counting cell numbers (Figure 7C). Consistent with impairment of ATF4 protein induction, TBL2-shRNA-expressing cells exposed to glc(−)/hypoxia stress for 12 hours exhibited delayed growth compared to control cells (Figures 7A). Likewise, the cell viability assay revealed that TBL2-shRNA-expressing cells become more sensitive to thapsigargin treatment than control cells (Figure 7B). In addition, we investigated cell growth by counting cell numbers of each shRNA-expressing cells after exposure to glc(−)/hypoxia stress for 12 hours. TBL2-shRNA-expressing cells showed a delayed growth compared to control cells while each cells proliferated at similar level in the case of non-exposure to stress (Figure 7C left and right). Thus, we found that TBL2 plays an important role in cell protection, especially under low nutrient conditions such as glc(−)/hypoxia.

## Discussion

Upon ER stress, activated PERK inhibits protein synthesis by phosphorylating eIF2α while it activates the transcription factor ATF4. ATF4, in turn, activates transcription of a variety of genes to adapt to stress conditions [26]. In this report, we have identified TBL2 as a protein selectively binding to phosphorylated form of PERK on the ER (Figures 1–3). Moreover, we showed TBL2 interacts with phospho-PERK via the 32-74aa region and also associates with eIF2α via the WD40 domain (Figure 4). Furthermore, we have provided evidence suggesting that TBL2 is involved in ATF4 induction and cell growth under stress conditions (Figures 5–7). These findings indicate that TBL2 is a potential regulator of the PERK pathway.

A limited number of effector or regulator of PERK pathway has been reported until now [27]–[29]. eIF2α is a well-characterized PERK substrate and a subunit of the heterotrimeric protein eIF2, which mediates the binding of methionyl-tRNA to the ribosome in a GTP-dependent manner [27], [28]. Phosphorylation of eIF2α inhibits the guanine nucleotide exchange activity of eIF2B by forming a complex with eIF2B [28], thus impairing the eIF2B-mediated recycling of eIF2 and leading to global inhibition of translation. Besides, an ER luminal molecular chaperone, GRP78/BiP has been reported to prevent PERK from autoactivation through binding to ER luminal region of PERK [29]. During ER stress, GRP78/BiP dissociates from PERK, resulting in allowing PERK to oligomerize and autoactivate [29]. On the other hand, TBL2 does not seem to modulate activation of PERK or PERK-mediated eIF2α phosphorylation. Indeed, TBL2 knockdown or its overexpression had little effects on phosphorylation of PERK and eIF2α and, in consistent, on general translational attenuation (Figures 2B, 5 and 6). Nevertheless, TBL2 knockdown impaired ATF4 induction at similar level to PERK knockdown under stress conditions (Figures 5 and 6). Therefore, TBL2 appears to regulate ATF4 induction in a manner that cannot be explained by the conventional PERK-mediated model.

A recent study identified a small compound ISRIB that selectively inhibits PERK branch, but not IRE1 and ATF6 branches [30]. ISRIB treatment results in suppressing ATF4 induction under ER stress without affecting eIF2α phosphorylation, which has been considered crucial for general translational attenuation and subsequent ATF4 induction [4], [30]–[32]. These observations may imply the presence of additional factors, like TBL2 shown here, that can be involved in PERK-mediated ATF4 induction. At present, it remains largely unknown about precise molecular mechanisms how TBL2 regulates ATF4 induction through the binding to PERK. Importantly, TBL2 also associated with eIF2α via the WD40 domain under both stress and non-stress conditions (Figure 4). Given that TBL2 is an ER-membrane protein, TBL2 would be able to form a complex with eIF2 on the ER. Conceivably, the interaction of the TBL2-eIF2 complex with PERK during ER stress may have a role in facilitating translation of specific targets by locally and spatially enhancing the availability of eIF2 on the ER. In this regard, it would be noteworthy that ATF4 mRNA can be distributed not only in the cytoplasm but also on the ER [33]. Further study on the TBL2 complex will be helpful to understand the mechanism of PERK-mediated gene expression under ER stress conditions.

We have shown, herein, that under ER stress, TBL2 is a new player that can be involved in ATF4 induction of the PERK pathway and can mediate cell survival. Similarly to TBL2 knockdown, depletion of ATF4 also has been reported to reduce cell survival under stress conditions such as glucose or amino acid deprivation, and hypoxia, which are cell conditions seen in solid tumor [24], [25]. These phenotypic similarities may imply that TBL2 is involved in tumor cell adaptation to poor nutrient conditions through induction of ATF4. ATF4 expression is not only induced by PERK activation but also by three other cytosolic eIF2α-kinases (PKR, HRI and GCN2), which are activated under viral infection or nutrient starvation conditions [34]–[36]. Given that TBL2 preferentially interacts with PERK, but not GCN2, each eIF2 kinase may have TBL2-like unique binding partner. Therefore, our study could provide important information to help elucidate how, under stress conditions, these eIF2α-kinases achieve the translation of specific mRNAs.

## Supporting Information

Figure S1 **PERK kinase domain is important to bind to TBL2.** 293T cells were transiently transfected with pFLAG-PERK-WT or pFLAG-PERK-DN and then were treated with 300 nM thapsigargin (Tg) for 1 h. The cell lysates were immunoprecipitated with anti-FLAG antibody and immunoblotted with the indicated antibody.
(TIFF)

Figure S2 **TBL2 knockdown has little effects on XBP1 splicing and GRP78 induction.** (A) Analysis of XBP1 transcript in TBL2 knockdown cells. The cells were transiently transfected with non-silencing siRNA, TBL2 siRNA or PERK siRNA. After 48 h, the cells were treated with 300 nM thapsigargin for the indicated times. To detect XBP1 mRNA splicing valiant, we amplified each cDNA using a specific primer pair that produces amplicon sizes of 441 bp (unspliced form) and 415 bp (spliced form). (B) The cells were transiently transfected with non-silencing siRNA, TBL2 siRNA or PERK siRNA. After 48 h, the cells were treated with 300 nM thapsigargin for the indicated times. Each lysate sample was subjected to immunoblot with the indicated antibody.
(TIFF)
