# Supplementary figures and images for: TBL2 Is a Novel PERK-Binding Protein that Modulates Stress-Signaling and Cell Survival during Endoplasmic Reticulum Stress
Source: PLoS One. 2014 Nov 13;9(11):e112761. doi: 10.1371/journal.pone.0112761 (PMC4231078; doi:10.1371/journal.pone.0112761)

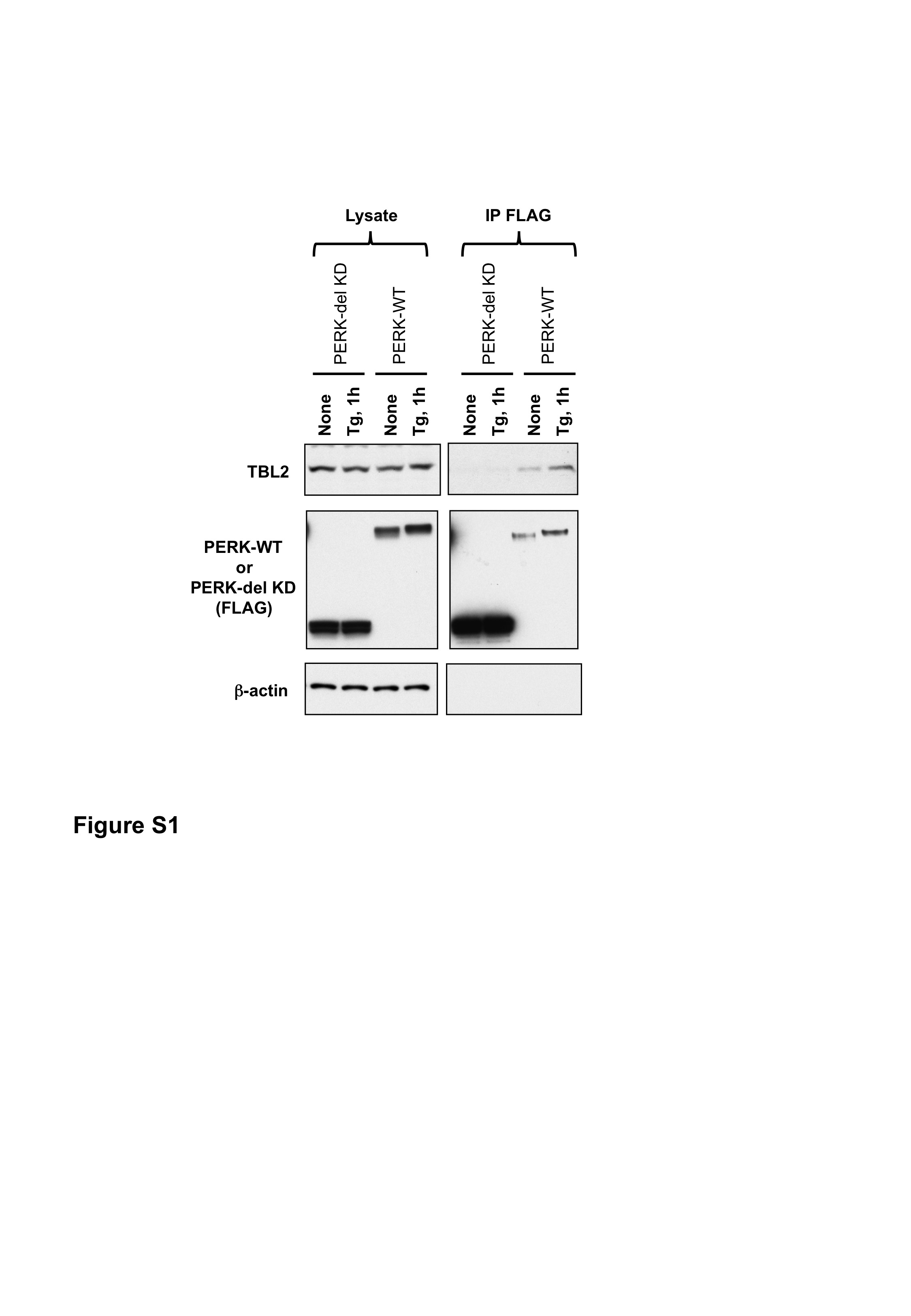

Supplement: Figure S1 — PERK kinase domain is important to bind to TBL2. 293T cells were transiently transfected with pFLAG-PERK-WT or pFLAG-PERK-DN and then were treated with 300 nM thapsigargin (Tg) for 1 h. The cell lysates were immunoprecipitated with anti-FLAG antibody and immunoblotted with the indicated antibody. (TIFF) [file pone.0112761.s001.tiff]

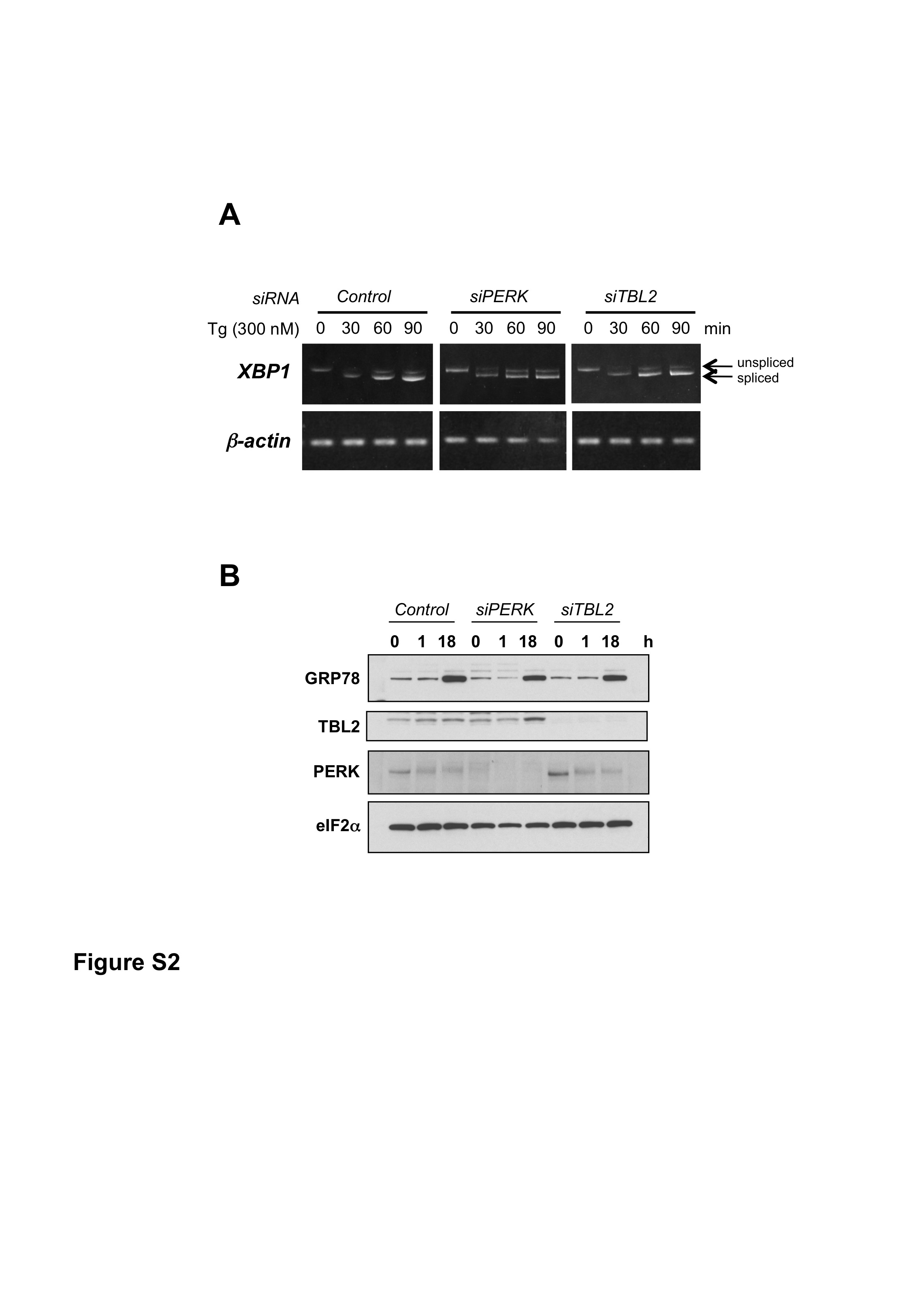

Supplement: Figure S2 — TBL2 knockdown has little effects on XBP1 splicing and GRP78 induction. (A) Analysis of XBP1 transcript in TBL2 knockdown cells. The cells were transiently transfected with non-silencing siRNA, TBL2 siRNA or PERK siRNA. After 48 h, the cells were treated with 300 nM thapsigargin for the indicated times. To detect XBP1 mRNA splicing valiant, we amplified each cDNA using a specific primer pair that produces amplicon sizes of 441 bp (unspliced form) and 415 bp (spliced form). (B) The cells were transiently transfected with non-silencing siRNA, TBL2 siRNA or PERK siRNA. After 48 h, the cells were treated with 300 nM thapsigargin for the indicated times. Each lysate sample was subjected to immunoblot with the indicated antibody. (TIFF) [file pone.0112761.s002.tiff]
